# Lessons from the integrated community case management (iCCM) Rapid Access Expansion Program

**DOI:** 10.7189/jogh.09.020101

**Published:** 2019-12

**Authors:** Salim Sadruddin, Franco Pagnoni, Gunther Baugh

**Affiliations:** 1 Global Malaria Programme, World Health Organization, Geneva Switzerland; 2 Director, TIPTOP Project, Barcelona Institute for Global Health, Barcelona, Spain; 3 Independent Consultant, Geneva, Switzerland (formerly with Global Malaria Programme, World Health Organization)

## Abstract

In 2012, the Government of Canada awarded a grant to the World Health Organization’s Global Malaria Programme (GMP) to support the scale-up of integrated community case management (iCCM) of pneumonia, diarrhoea and malaria among children under 5 in sub-Saharan Africa under the Rapid Access Expansion Programme (RAcE). The two main objectives of the programme were to: (1) Contribute to the reduction of child mortality due to malaria, pneumonia and diarrhoea by increasing access to diagnostics, treatment and referral services, and (1) Stimulate policy updates in participating countries and catalyze scale-up of integrated community case management (iCCM) through documentation and dissemination of best practices. Based on the results of the implementation research and programmatic lessons, this collection provides evidence on impact and improving coverage of iCCM in routine health systems, and opportunities and challenges of implementing and sustaining delivery of iCCM at scale.

After the publication of the WHO and UNICEF joint statement on iCCM, countries started implementing small-scale iCCM projects funded by global funding agencies [1]. The American Journal of Tropical Medicine and Hygiene 2013 supplement on “Evidence for the implementation, effects, and impact of the integrated community case management strategy to treat childhood infection” and the iCCM evidence symposium and subsequent publications of articles in the Journal of Global Health 2014 supplement provided an impetus for scaling up iCCM [2,3]. However, the donor-supported initiatives to introduce iCCM through NGOs in many countries, oftentimes bypassing the Ministries of Health, raise questions on continuity and sustainability of service delivery by community health workers. According to Daelmans et al., a persistent challenge to scaling up iCCM has been understanding prerequisites for successful iCCM implementation at national scale [4]. The multi-country RAcE Programme was thus set up to strengthen the evidence base for iCCM best practices and sustainability and to generate knowledge for policy-makers and practitioners to accelerate access to care [5].

The WHO’s RAcE Programme governed by an International Steering Group selected civil society organizations (CSO) to receive grants to support implementation of iCCM in the Democratic Republic of Congo, Malawi, Mozambique, Niger and two States in Nigeria. The CSOs were selected through a transparent review process by an independent body, the Project Review Panel (PRP), consisting of members with expertise on iCCM and health systems strengthening. In each country RAcE initiative was implemented under the leadership of Ministry of Health, with iCCM technical committees chaired by the MOH providing technical support and programme oversight.

Over 1.49 million children were provided access to life saving treatments for malaria, pneumonia and diarrhoea through training and equipping of more than 8200 Community Health Workers (CHWs) in the 5 countries. In each country the programme was at scale, covering all of Tanganyika Province in DRC, 8 Districts in Malawi, 4 Provinces in Mozambique, 4 Districts in Niger, and two States in Nigeria. Four years of implementation resulted in diagnosis and treatment of over 8.24 million clinical cases of malaria, pneumonia and diarrhoea by community health workers.

The implementing partners were encouraged to include implementation research in their proposals. Implementation research studies were embedded in the programmes, and placed under the overall strategic guidance of the country level steering committee headed by the Ministry of Health. WHO undertook the role of technical support and oversight. A dissemination meeting to share the results of implementation research and best practices with policymakers and implementers was organized in Abuja, Nigeria in 2014. Following the Abuja meeting the study investigators, and CSO and MOH technical staff were encouraged to develop manuscripts for sharing the research results and programmatic lessons with neighboring countries and globally.

This supplement presents results of the implementation research and programmatic learning in the context of the RAcE programme in the five countries. The articles are structured around five themes: **Impact of iCCM on care seeking and treatment and child mortality; sustainability; monitoring and health information systems; challenges in mature iCCM programmes; and tools and approaches to improve quality of care.**

With regards to impact om mortality, Prosnitz et al. present the results of RAcE programme evaluation to demonstrate plausible contribution of RAcE to any changes in treatment coverage and mortality in RAcE supported programme areas. The Lives Saved Tool was used to estimate changes in child mortality in RAcE sites. Under-five mortality declined in all six RAcE sites, with an overall average decline of 10 percent.

Oresanya et al. and Isiguzo et al. in their articles demonstrate the effect of introducing iCCM in increasing care seeking and treatment from CHWs in two geographically and culturally diverse states in Nigeria. Their analysis demonstrates how bringing quality care closer to home improves overall care seeking and also shifts care seeking from public sector facilities that are not accessible or provide poor quality care, as well as from for-profit retail drug outlets. Maintaining the improvements in health outcomes beyond the introductory phase requires a structured, participatory planning approach. Yourkavitch et al describe a facilitated sustainability planning process to identify and address health systems factors that influence the sustainability of iCCM services, along with planning for transition from an externally supported implementation to a national system. While, Alegbeleye et al describe the process of community engagement and mobilization in Niger State, and how it fostered ownership and service uptake.

In regard to monitoring and health information systems, Moonzwe Davis et al. and Yourkavitch et al. in their articles describe a unique approach to assess iCCM reporting systems and data quality and how iCCM data quality assessments can serve as a collaborative and evidence-based activity to influence discussions of data quality and stimulate HMIS strengthening efforts.

Two articles from Malawi and one from Mozambique highlight the challenges faced by mature programmes implemented at scale. Zalisk et al. present the findings from the household surveys in Malawi. The article highlights factors that influence communities’ trust and confidence in CHWs and resulting effect on care seeking from CHWs. The article by Guenther et al. on introduction of home visits for newborn and pregnant women in the CHW service delivery package in Malawi demonstrates the need for thorough assessment of CHW capacity and workload, and community acceptability before introduction of a new intervention or delivery strategy. Källander et al. present the limitations of CHW training and deployment on child mortality. This has to be accompanied by increasing community awareness to seek care promptly, improved referral linkages and availability of CHWs when a child falls ill. The challenges faced by the multi-purpose CHWs in Malawi and Mozambique, who service multiple villages with a variety of curative, preventive and promotive activities, or spending significant amount of time in the facility demonstrates the limits of this cadre. These CHWs are overloaded, and programmes keep identifying more interventions and tasks for them as part of an expanding community platform. Interventions to be delivered by CHWs must be prioritized based on an objective evaluation in terms of positive or negative synergistic effects at the CHW level.

In regard to tools and approaches for improving quality of care delivered by CHWs, Langston et al. demonstrate the improvements that can be made through streamlining existing tools and CHW training strategy. Peart Boyce et al. and Zakus et al. present results of studies assessing effect of smart phones with mHealth applications on quality of care delivered by the CHWs in Malawi and Niger, respectively. Both studies observed that mHealth applications improved adherence to the iCCM protocol for assessing sick children and classifying illness by CHWs, with limited or no effect on treatment. Considering additional costs, logistics and national capacity to manage the mHealth system, questions around its viability remain.

The diverse set of articles in the supplement along the spectrum of implementation and different health systems provide valuable lessons for implementing iCCM and important reflections for scale-up within the health system. They also highlight the importance of carrying out implementation research as part of program implementation in order to maximize gains in public health knowledge.
